# Curve-Based Classification Approach for Hyperspectral Dermatologic Data Processing

**DOI:** 10.3390/s21030680

**Published:** 2021-01-20

**Authors:** Stig Uteng, Eduardo Quevedo, Gustavo M. Callico, Irene Castaño, Gregorio Carretero, Pablo Almeida, Aday Garcia, Javier A. Hernandez, Fred Godtliebsen

**Affiliations:** 1 Department of Education and Pedagogy, UiT the Arctic University of Norway, 9019 Tromsø, Norway; 2 Institute for Applied Microelectronics, Universidad de Las Palmas de Gran Canaria, 35016 Las Palmas de Gran Canaria, Spain; eduardo.quevedo@ulpgc.es (E.Q.); gustavo@iuma.ulpgc.es (G.M.C.); 3 Department of Dermatology, Hospital Universitario de Gran Canaria Doctor Negrín, 35016 Las Palmas de Gran Canaria, Spain; irenecastano@hotmail.com (I.C.); gcarher@gobiernodecanarias.org (G.C.); 4 Department of Dermatology, Complejo Hospitalario Universitario Insular-Materno Infantil, 35016 Las Palmas de Gran Canaria, Spain; pjalmmar75@gmail.com (P.A.); jhersant@gobiernodecanarias.org (J.A.H.); 5 Department of Electromedicine, Complejo Hospitalario Universitario Insular-Materno Infantil, 35016 Las Palmas de Gran Canaria, Spain; agarcia@iuma.ulpgc.es; 6 Department of Mathematics and Statistics, UiT the Arctic University of Norway, 9019 Tromsø, Norway; fred.godtliebsen@uit.no

**Keywords:** hyperspectral, curve fit, statistical discrimination, melanoma, benign, malignant

## Abstract

This paper shows new contributions in the detection of skin cancer, where we present the use of a customized hyperspectral system that captures images in the spectral range from 450 to 950 nm. By choosing a 7 × 7 sub-image of each channel in the hyperspectral image (HSI) and then taking the mean and standard deviation of these sub-images, we were able to make fits of the resulting curves. These fitted curves had certain characteristics, which then served as a basis of classification. The most distinct fit was for the melanoma pigmented skin lesions (PSLs), which is also the most aggressive malignant cancer. Furthermore, we were able to classify the other PSLs in malignant and benign classes. This gives us a rather complete classification method for PSLs with a novel perspective of the classification procedure by exploiting the variability of each channel in the HSI.

## 1. Introduction

Hyperspectral (HS) imaging (HSI) combines conventional imaging and spectroscopy methods in a single imaging technique providing both spatial and spectral information of the captured display [1,2]. It is also a fitting method for medical applications due to its non-invasive, non-ionizing, and label-free nature [3]. Dermatology is one of the medical fields where HSI have shown its potential as an appropriate imaging technique [4].

Currently, the most common form of cancer, with more than 1.3 million new cases worldwide in 2018, is skin cancer [5]. There are several types of skin lesions. Pigmented skin lesions (PSLs) contain a wide variety of types, including cancerous and non-cancerous PSLs [6]. There are two types of PSLs depending on the type of growth of the tissue: benign and malignant. Nevus, which is benign, has a slow growth rate and is noncancerous, while e.g., melanomas, which is malignant, are invasive and potentially metastatic tumors [6]. Other types of skin cancer produced by different types of cells include squamous cell carcinoma and basal cell carcinoma. However, melanomas are much more dangerous than other types of skin cancer, and an early detection of this skin lesion can be extremely important in improving the patient survival [7]. The diagnosis of PSLs is performed by dermatologists employing their naked eye or dermascopic cameras, which enhance the morphological visualization of the PSL. After the inspection, an analysis of the lesion following the ABCDE (Asymmetry, Border irregularity, Color, Diameter and Evolving size, shape, or color) rule is applied in order to establish a preliminary diagnosis [7]. A suspicious lesion will accordingly get a histopathological analysis carried out to determine the definitive diagnosis. These diagnostic tools based on conventional imaging have been used to help dermatologists in preliminary diagnosis. However, conventional imaging has limitations that could be surpassed by the enriched spectral information provided by the HSIs.

HSI technology has already been explored as a target technology to aid in PSLs diagnosis. A recent study by Leon et al. showed promising results in discriminating PSLs by random forests and artificial neural networks [8]. Another recent study by Hosking et al. used digital dermascopy HSI with machine learning methods in order to detect melanomas [9]. Kazianka et al. used HSIs to differentiate between normal skin, melanomas, and moles [10]. They used unsupervised approaches to segment the images and moreover evaluated the performance of several supervised classifiers aimed to retrieve the diagnosis of each pixel. The results indicated that it was possible to differentiate melanomas from moles with high specificity and sensitivity. In another study, Nagaoka et al. [11] collected a dataset for discriminating between melanomas and other PSLs, evaluating the statistical significance of an index defined to this end. Tomatis et al. used a classifier based on a neural network model [12]. There have also been developed some commercial systems as SIAscope/SIAscopy [13] and MelaFind [14,15,16]. MelaFind is being used in several studies. In [15], Elbaum et al. conduct a leave-one-out cross-validation procedure using a database composed of 183 melanocytic nevus and 63 melanomas. Monheit et al. [16] applied it in a multicenter study. At last, Song et al. performed a paired comparison between MelaFind and a reflectance confocal microscopy system to differentiate between melanoma and non-melanoma PSLs by comparing MelaFind with a confocal microscopy system. In the study by Stamnes et al., they discriminate between malignant and benign PSLs, i.e., no specific melanoma detection [17].

The current default method of data analysis (DA) is deep learning (DL). DL emerged as a competing DA method around 2009 and solves a wide range of problems exemplified by the versatile TensorFlow-package [18]. DL is also applied in the HSI field. However, DL often needs a huge amount of data in order to train the parameters enough in the neural network [19]. Thus, in the proposed method, this presents a difficult problem due to the lack of data. We are pursuing this problem by an ongoing data collection project, aiming for future applications of DL systems.

Most of the research in the use of HSI for skin analysis is focused on the automatic diagnosis of PSLs. Clearly, a successful methodology of this type would be an extremely useful decision support tool for general practitioners (GPs) and dermatologists. In our contribution in this direction, we were able to find clear patterns for the melanoma PSLs, the other malignant PSLs and the benign PSLs. In the novel exploitation of the variability of each channel in the HSI cubes, we made curves of the mean and standard deviation of sub-images in these channels. Furthermore, using these curves and their inherent patterns, we were able to discriminate between malignant and benign PSLs, but also between the aggressive melanoma PSL and the others.

The outline of the paper is as follows. First, we describe the developed dermatological HSI acquisition system, and thereafter, we describe the datasets we are using in this curve-based classification method. Next, we point out how the data are pre-processed and labeled before they are used as input in our new classification method, which consists of a training, validation, and testing phase.

## 2. Materials and Methods

### 2.1. Hyperspectral In Vivo Dermatologic Data

The system used to acquire the HS dermatologic images is described in [20], using the same database as in Leon et al. [8]. The database consists of 76 images of PSLs from 61 patients: 36 cancerous and 40 noncancerous. The dataset is divided into a training set, a test set, and a validation set. The training set is subsequently divided into melanoma PSLs, malignant PSLs, and benign PSLs. Each mentioned set is mutually exclusive. The acquisition system is composed of an HS snapshot camera able to capture HS images in the very near infrared (VNIR) range, between 450 and 950 nm, with a spatial resolution of 50 × 50 pixels and 125 spectral bands. This acquisition system employs a customized dermascopic contact structure and a halogen source light (150 W) coupled to a fiber optic ring light guide for cold light emission. The effective capturing area of the system is 12 × 12 mm, and the acquisition time is lower than 1 s. The system was applied to create an HS database composed of 76 images of PSLs from 61 patients. The data acquisition was done at the Hospital Universitario de Gran Canaria Doctor Negrín and Complejo Hospitalario Universitario Insular-Materno Infantil (Spain). The research protocol and consent procedures were approved by the Comité Ético de Investigación Clínica-Comité de Ética en la Investigación (CEIC/CEI) at the University Hospital Doctor Negrin.

### 2.2. Curve-Based Classification Approach

In the pre-processing step, a calibration is done of the HSI using the white and dark reference images following Equation (1), where *CI* is the calibrated image, *RI* is the raw image obtained from the HS camera, and *DI* and *WI* are the dark and white reference images, respectively. The white reference image is obtained capturing an image of a white reference tile that reflects 99% of the incident light. The dark reference image is captured by keeping the tap in the lens of the camera.

(1) $$CI=\frac{RI-DI}{WI-DI}$$

Finally, the data is normalized in the range [0,1] to avoid differences in the spectral signature intensities caused by possible different illumination conditions.

### 2.3. Region of Interest Curves

The region of interest (ROI) was obtained through the extraction of 7 × 7 sub-images for all the channels. Subsequently, the mean (x-coordinate of the curves) and standard deviation (y-coordinate of the curves) of these ROIs from each HS channel were plotted against each other, thus creating ROI curves for each HSI cube. These curves were rather different, some almost linear, other more convoluted. Thus, we applied a morphological dilation filter to the more complex curves to ease the interpretation and subsequently use these filtered curves in function fits. Morphological dilation is a common way to enhance or change digital images in some way. Thus, often used in bridging gaps, our morphological filter can be seen as an inverse dilation, a pruning.

There were some issues of this method that needed to be addressed. In particular, how the ROIs are chosen. So, the first issue is where to collect this ROI in the HSI, i.e., which coordinates to choose? To shed some light on this, we established some criteria for each of the classes of PSLs and further picked the ROIs best suited for creating the curves with adequate classification properties. In each channel, we started from (*x,y*) = (16, 16) and went to (*x,y*) = (35, 35). This gave us 20 × 20 = 400 HS ROI curves for each PSL HSI cube collected from different locations. However, with some overlap and since each ROI area is 7 × 7 pixels, we cover the HSI from the start at (*x,y*) = (13, 13) to the end at (*x,y*) = (38, 38): thus, an area of 26 × 26 = 676 pixels. We tried a wider area, but the results did not improve, so we kept this size due to computational cost. Thus, we tested how the ROI extraction affected the mean square error (MSE) of the curves chosen by suitable criteria for the melanoma as well as the other malignant and benign PSLs.

The CPU used to run the code was an Intel Xeon 2.8 GHz running on a 16 GB RAM computer.

#### Curve-Fitting

By trying to classify the PSLs through fits of the ROI curves, all the HSI ROI curves were fitted by a quartic (fourth degree) polynomial function, where the coefficients are found through the nlinfit function of MATLAB^®^, which minimizes the weighted least squares equation,

(2) $$\sum_{i=1}^{n}w_{i}{\left(y_{i}-f\left(x_{i},b\right)\right)}^{2}$$

where the weights were chosen through a weight function, *w*(*y*) = 1/(0.011 + 0.011*y*)^2^ and *n* = 125, the number of data points. Further is *f*(*x*, *b*) = *b*_0_ *x*^4^ + *b*_1_ *x*^3^ + *b*_2_ *x*^2^ + *b*_3_ *x* + *b*_4_, the quartic regression function, with parameters *b_j_*, *j* = *0*,…,4. Then, the nlinfit-function uses the iteratively reweighted least-square algorithm, which is designed to deal with outliers [21]. Furthermore, the use of a quartic polynomial with a weight function proved to be the best option with regard to flexibility and robustness to outliers. We also tested higher degree polynomial functions; however, they were prone to overfitting.

**Figure 1:**
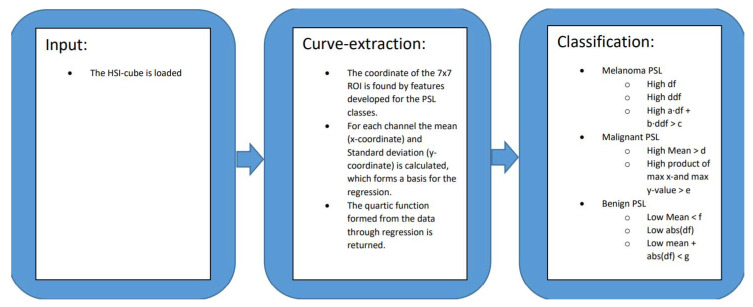
Schematic overview of the algorithm.

Figure 1 gives an overview of the algorithm. The constants a, b, c, d, e, f, and g are found though the training phase. The features are as follows: df (first-order derivative), ddf (second-order derivative), totm = a·df + b·ddf, mabdf = mean + abs(df) and mean.

## 3. Results

Here, we will represent the obtained experimental results. We start the representation of the training phase.

### 3.1. The Training Phase of the Method

**Figure 2:**
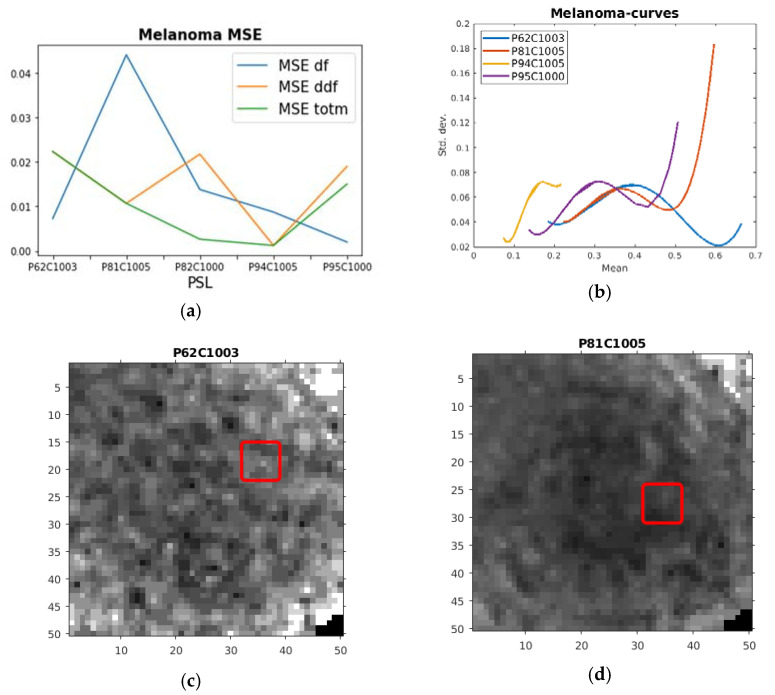

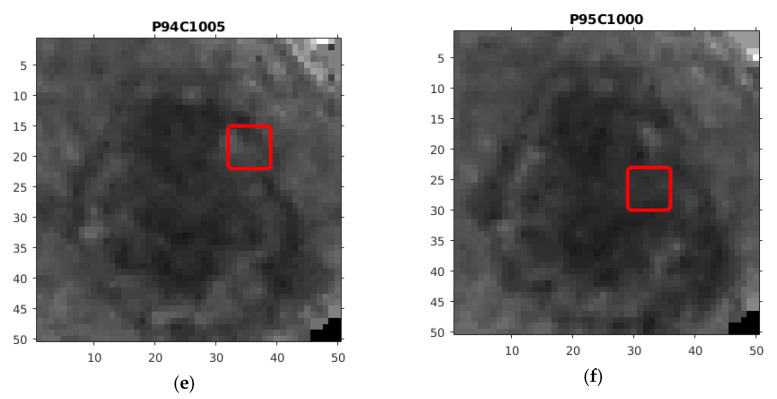
(**a**) Plot of mean square error (MSE) first-derivative feature (MSE df), second-derivative feature (MSE ddf) and sum feature (MSE totm) for the hyperspectral image (HSI) ROI curves from the training melanoma PSLs. (**b**) Plot of quartic polynomial fits of melanoma HSI ROI curves chosen according to the low MSE totm. (**c**) The corresponding ROI in red drawn on the HSI for one of the channels used in the creation of the HSI ROI curve from the P62C1003 PSL. (**d**) The corresponding ROI in red drawn on the HSI for one of the channels used in the creation of the HSI ROI curve from the P81C1005 PSL. (**e**) The corresponding ROI in red drawn on the HSI for one of the channels used in the creation of the HSI ROI curve from the P94C1005 PSL. (**f**) The corresponding ROI in red drawn on the HSI for one of the channels used in the creation of the HSI ROI curve from the P95C1000 PSL.

As mentioned above, we acquired an ROI from each channel of the HSIs, where the curves were formed through the calculation of the mean and standard deviation of the ROIs from each channel. First, we investigated the melanoma PSLs. The features coming most natural from earlier experiments are a high mean first-order derivative (Max df) and a high mean curvature given by the mean of the second derivative (Max ddf). However, during the training phase, we found that a high sum of mean df (multiplied by 1, a in Figure 1), mean ddf (multiplied by 0.1, b in Figure 1), and the derivative at the maximum value gave the most successful feature (Max totm > 2.86, c in Figure 1). In Table 1 and Figure 2a, we can observe that this feature produced rather good fits according to the MSEs: the MSE ddf following this measure and the MSE df lower for two melanomas. Thus, all the plots of the HSI ROI curves from the melanoma PSLs are from the fits of the Max totm curves. Moreover, the recurring theme in Figure 2c–f is that all the HSI ROIs came from the interior and/or near the edges of the melanomas. In Table 1, we can also observe the most distinct outlier, the P82C1000 PSL, which did not have the same characteristics as the other melanomas, behaving more similar to the malignant PSLs. In Figure 2a, we can observe that this PSL is the one with an MSE ddf diverging most clearly from the MSE totm.

**Figure 3:**
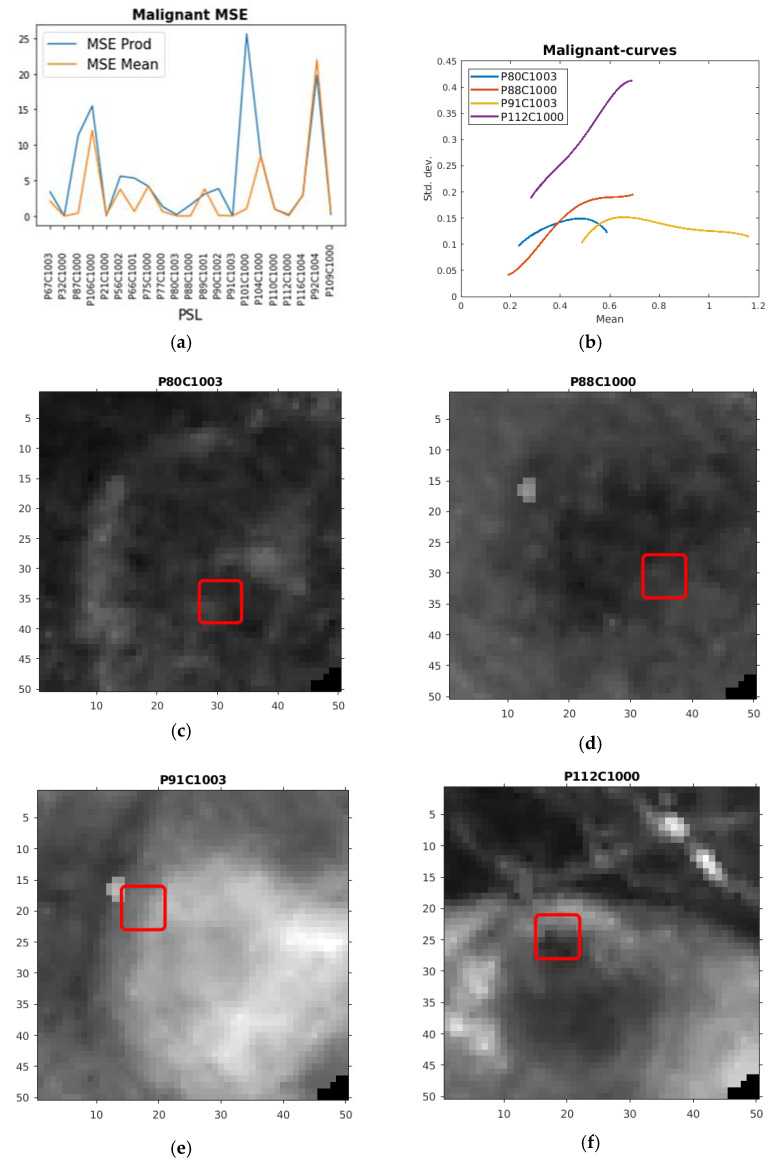
(**a**) Plot of MSE product of max x and max y (MSE Prod) and maximum mean (MSE Mean) for the fitted HSI ROI curves from the malignant PSLs. (**b**) Plot of quartic polynomial fits of malignant HSI ROI curves chosen according to low MSE mean. (**c**) The corresponding ROI in red drawn on the HSI for one of the channels used in the creation of the HSI ROI curve from the P80C1003 PSL. (**d**) The corresponding ROI in red drawn on the HSI for one of the channels used in the creation of the HSI ROI curve from the P88C1000 PSL. (**e**) The corresponding ROI in red drawn on the HSI for one of the channels used in the creation of the HSI ROI curve from the P91C1003 PSL. (**f**) The corresponding ROI in red drawn on the HSI for one of the channels used in the creation of the HSI ROI curve from the P112C1000 PSL.

Regarding the malignant PSLs, we inferred based on earlier experiments that the HSI ROI curves with a high mean (Max Mean > 0.05, d in Figure 1) or a high product of max x and max y (Max Prod > 0.395, e in Figure 1) from the fitted curve are plausibly originating from this type of PSL. This proved successful for some of the HSI ROI curve fits from the malignant PSLs. As seen in Table 2 and Figure 3a, this was the most difficult type PSL to fit, giving rise to rather high MSEs. The Max Mean was the best feature accordingly. Some of the curves with least MSE Mean are plotted in Figure 3b, where all have means lying above the standard deviation (y-value) of 0.05 (d in Figure 1). From these HSI ROI mean standard deviation curves, the most complex data occurred—thus, as mentioned above, giving rise to rather high MSEs. The corresponding ROIs of the plotted malignant curves are also here lying on the inside or at the edge of PSLs, as seen in Figure 3c–f.

**Figure 4:**
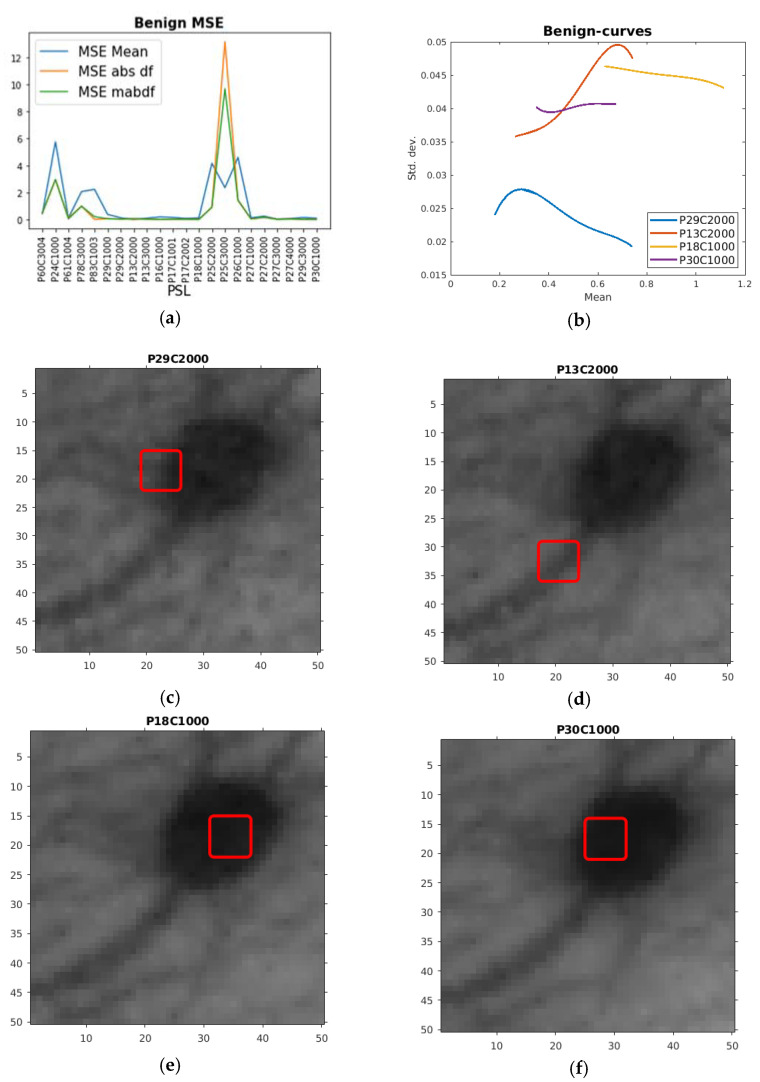
(**a**) Plot of MSE mean feature (MSE Mean), absolute value of the first derivative feature (MSE abs df), and the sum of these (MSE mabdf) for the HSI ROI curves from the benign PSLs. (**b**) Plot of quartic polynomial fits of benign HSI ROI curves chosen according to low MSE abs df. (**c**) The corresponding ROI in red drawn on the HSI for one of the channels used in the creation of the HSI ROI curve from the P29C2000 PSL. (**d**) The corresponding ROI in red drawn on the HSI for one of the channels used in the creation of the HSI ROI curve from the P13C2000 PSL. (**e**) The corresponding ROI in red drawn on the HSI for one of the channels used in the creation of the HSI ROI curve from the P18C1000 PSL. (**f**) The corresponding ROI in red drawn on the HSI for one of the channels used in the creation of the HSI ROI curve from the P30C1000 PSL.

The benign PSLs HSI ROI curves proved easier to fit than the malignant ones. From earlier experiments, two useful features came to be low mean (Min Mean < 0.05, g in Figure 1), low absolute value first order derivative (Min abs df), and the sum of these (Min mabdf < 0.109, f in Figure 1). This proved rather successful with a few exceptions, as can be observed in Table 3 and Figure 4a, with Min abs df and Min mabdf as similar rather successful features. In Figure 4b, four of the curves with lowest MSE abs df are plotted with their corresponding placements of the ROIs given in Figure 4c–f. We can here see that for these PSLs, the ROIs are somewhat different placed than for the melanomas and malignant ones—not all inside the PSLs, but at some sort of feature, nevertheless.

#### Classification

From the training phase, we inferred that the classification procedure can consist of two consecutive steps.

First, we identify the curves where the first derivative increases, possibly converging at maximum value of the HSI ROI curves mean. Additionally, the curves with high mean positive curvature and less double inflexion points were also identified as melanomas. In addition, the sum df + 0.1ddf proved successful (totm).

Then, we find the two clusters of the rest of the curves, such that they can be classified as benign or malignant. This is mainly done by choosing what curves have mean above y = 0.05 (malignant) and below y = 0.05 (benign). For the benign curves, an additional useful feature was the sum of the mean of the fitted curve and the mean absolute value of the first-order derivative in each point (mabdf).

### 3.2. Validation and Test Experiment

**Figure 5:**
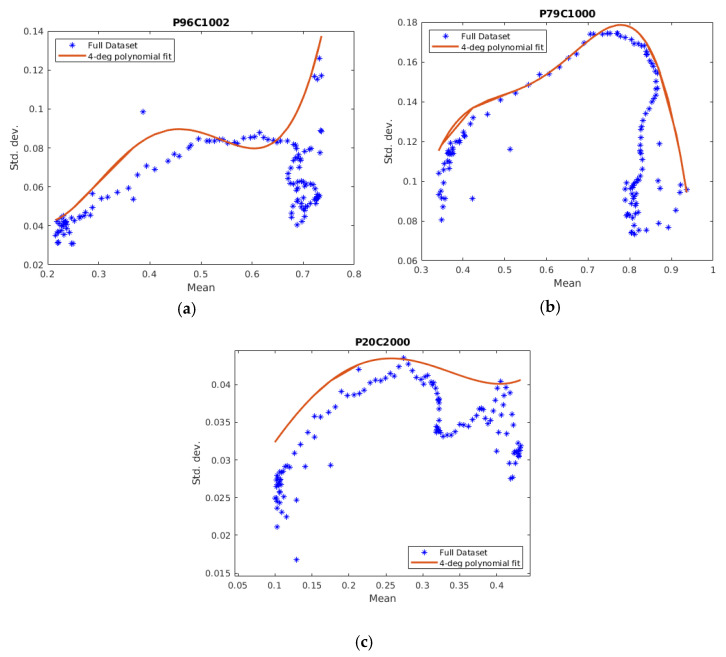
Examples of the fitted curve plots with data points from the validation phase: (**a**) Plot of a quartic polynomial fit of a melanoma HSI ROI curve and the data points. (**b**) Plot of a quartic polynomial fit of a malignant HSI ROI curve and the data points. (**c**) Plot of a quartic polynomial fit of a benign HSI ROI curve and the data points.

We also performed an experiment in order to validate the training phase with the same type of ROI curves as in the training part but different PSLs. This resulted in the usual 125 points mean and standard deviation curve; then, a quartic polynomial function was fitted, as mentioned earlier. Based on the features developed in the training phase, then, we were able to first discriminate between melanomas and the rest of the PSLs, where a typical melanoma fit is given in Figure 5a, and then discriminate between malignant and benign PSLs. Examples of their respective fits given in Figure 5b,c.

When looking at the results, we denote correctly classifying a malignant PSL as a true positive (tp) and correctly classifying a benign PSL as a true negative (tn). Furthermore, falsely classifying a malignant PSL is denoted as a false positive (fp) and at last, falsely classifying a benign PSL is denoted as a false negative (fn). By using the validated classification criteria, we were able to test the method with a test set. The results are given in Table 4, where the P23C1001 gave a false positive due to some regression difficulties regarding outliers.

The accuracy of this proposed method is 90%. The sensitivity is 100% and the specificity is 80%. Considering the melanoma discrimination, both sensitivity and specificity are 100%. In Table 5 results from other researchers are provided.

From Table 5, we can discern that most research studies have been discrimination of the aggressive melanoma PSLs, where Nagaoka et al. [11] and Leon et al. [8] had the highest sensitivity and specificity. Stamnes et al. [17] also had high such measures; however, these were only discriminating between benign and malignant PSLs. Thus, the sensitivity and specificity in the method developed here is comparable to the others.

The works listed in Table 5 mostly use an array of machine learning methods, while the proposed method only use a regression method, making it a simpler approach than the others.

The computation time was on average 0.16 s for each PSL. However, including the best ROI search, this jumps to 70 s. Thus, it is a rather slow algorithm due to rather complex calculations. Compared to the previous work in [8], in which six of the 10 patients were correctly identified with an average execution time of 0.5 s, the proposed methodology identifies nine out of 10 patients with an average of 160 s. However, this is only the core algorithm; an efficient segmentation algorithm has to be included also, raising the execution time.

## 4. Limitations and Future Directions

Further studies must be conducted in order to improve the limitations in this research. The first limitation is the rather low number of samples in particular for the melanoma class. In addition, in the benign and malignant class, it would have been desirable of an increase of samples. However, the number of samples was enough to find and validate the patterns for each class of PSLs considered here. Another limitation is the computing time. However, this will be decreased by the automatic choosing of ROIs by segmentation of the PSLs, which have several known potential solutions. We will also try to improve the regression of the curves by further experimentation of the weight function or other types of regression. The developed system in this paper is at an experimental level; however, it has the potential to assist in the diagnosis of skin cancer and reduce the number of biopsies relating to this.

## 5. Conclusions

The work presented here shows that HSIs can be used to discriminate between melanoma, malignant, and benign PSLs as a potential future diagnostic tool for dermatologists or GPs. The proposed curve-based method is able to do this discrimination by using the inherent patterns of these curves. The presented method was able to correctly classify nine out of 10 in the test set, identifying all cancerous cases accurately (100% of sensitivity). Thus, it is a rather good result. In addition, in the test phase, it had a sensitivity of 80% overall and 100% for the melanomas. Since this method is relatively experimental, we will improve it further in order to increase its accuracy and lower its computation time. The limitation of the proposed method is mainly the lack of data, which is remedied in an ongoing project of data collection. The merits of the proposed method is the utilization of the richer structure provided by the HSI technology in a novel way.

## Figures and Tables

**Table 1:** The results for the region of interest (ROI) investigation for the melanoma pigmented skin lesions (PSLs) with the corresponding curves with maximum mean first derivative, Max df, maximum curvature, Max ddf, maximum weighted sum of mean df, mean ddf and df at the maximum point, Max totm and the corresponding MSEs of the chosen curves.

PSL	Max df	Max ddf	Max totm	MSE df	MSE ddf	MSE totm
P62C1003	0.19959	5.2261	1.2716	0.00729	0.022353	0.022353
P81C1005	0.90111	14.465	5.0123	0.044138	0.010691	0.010691
P82C1000	0.17383	–0.1158	−0.3373	0.01381	0.021781	0.0026241
P94C1005	1.1388	45.812	5.0485	0.008742	0.001214	0.0012136
P95C1000	0.60491	19.092	3.3212	0.001983	0.019006	0.015033

**Table 2:** The results for the ROI investigation for the malignant PSLs with the corresponding curves with maximum product of max x times max y, Max Prod, maximum mean, Max Mean, and the corresponding MSEs of the chosen curves.

PSL	Max Prod	Max Mean	MSE Prod	MSE Mean
P67C1003	0.20058	0.15051	3.4133	2.0846
P32C1000	0.05982	0.054024	0.02244	0.006436
P87C1000	0.33499	0.2143	11.323	0.41494
P106C1000	0.9194	0.35846	15.474	11.997
P21C1000	0.13709	0.11865	0.039819	0.16209
P56C1002	0.73114	0.17111	5.6309	3.7864
P66C1001	0.17669	0.11542	5.3595	0.64848
P75C1000	0.36819	0.18003	4.1902	4.234
P77C1000	0.31525	0.15402	1.3532	0.65383
P80C1003	0.094159	0.13404	0.2015	0.033497
P88C1000	0.16356	0.14231	1.5906	0.038208
P89C1001	0.23628	0.15301	3.1098	3.8229
P90C1002	0.33155	0.19675	3.872	0.087464
P91C1003	0.1763	0.12843	0.039625	0.039625
P101C1000	0.63537	0.24154	25.639	1.0538
P104C1000	0.77046	0.20304	8.3724	8.3684
P110C1000	0.18187	0.097568	0.96777	0.96777
P112C1000	0.34958	0.33396	0.19203	0.045852
P116C1004	0.26348	0.14593	2.9284	2.9284
P92C1004	1.4734	0.28289	19.805	21.99
P109C1000	0.38193	0.23467	0.23374	0.48969

**Table 3:** The results for the ROI investigation for the benign PSLs with the corresponding curves with minimum mean, Min Mean, minimum absolute value of the first derivative Min abs df, minimum sum of mean and absolute value of the first derivative Min mabdf, and the corresponding MSEs of the chosen curves.

PSL	Min Mean	Min absdf	Min mabdf	MSE Mean	MSE absdf	MSE mabdf
P60C3004	0.035875	0.04211	0.078126	0.43249	0.49182	0.49182
P24C1000	0.047758	0.13751	0.20262	5.7292	2.9709	2.9709
P61C1004	0.037392	0.019541	0.070525	0.11543	0.12084	0.049766
P78C3000	0.054088	0.14207	0.22912	2.0738	0.98825	0.98825
P83C1003	0.053259	0.051972	0.15544	2.2384	0.006806	0.20734
P29C1000	0.017986	0.037797	0.067169	0.37593	0.056122	0.056122
P29C2000	0.016499	0.028643	0.050963	0.12963	0.029577	0.029577
P13C2000	0.018738	0.025028	0.068659	0.004459	0.018544	0.073912
P13C3000	0.020795	0.058914	0.086964	0.097557	0.033792	0.020257
P16C1000	0.017759	0.049908	0.073101	0.18981	0.013001	0.013001
P17C1001	0.019861	0.021036	0.055137	0.15354	0.013007	0.028137
P17C2002	0.023828	0.012184	0.047559	0.079709	0.020721	0.018856
P18C1000	0.023887	0.010399	0.054889	0.11762	0.007339	0.0073387
P25C2000	0.053336	0.21251	0.27597	4.1742	0.91204	0.91204
P25C3000	0.057866	0.13919	0.22013	2.3664	13.174	9.6771
P26C1000	0.050246	0.18733	0.2605	4.5807	1.4319	1.4319
P27C1000	0.023408	0.052952	0.08173	0.12196	0.039168	0.047658
P27C2000	0.020774	0.045139	0.069434	0.24855	0.16042	0.16042
P27C3000	0.02075	0.058085	0.086034	0.029003	0.029591	0.016113
P27C4000	0.019193	0.026195	0.051928	0.070661	0.037739	0.037739
P29C3000	0.017297	0.042738	0.068037	0.14874	0.01073	0.022491
P30C1000	0.02094	0.00574	0.045026	0.09903	0.001885	0.0032779

**Table 4:** The PSLs in the test set and their classification correctness.

PSL	Correctness
P100C1000	tp
P23C1001	fp
P102C1000	tp
P28C1000	tn
P107C1003	tn
P69C1003	tp
P13C1000	tn
P74C1002	tp
P14C1000	tn
P97C1004	tp

**Table 5:** Comparison with results from other studies. (* Sensitivity for melanoma detection. ¥ Only reported sensitivity for three melanoma lesions. α Only reported sensitivity for four melanoma lesions).

References	Patients	Images	Bands	Range (nm)	Sensitivity (%)	Specificity (%)
Tomatis et al. [12]	1278	1391	15	483–950	80.4 *	75.6
Kazianka et al. [10]	-	310	300	-	95 *	-
Moncrieff et al. [13]	311	348	8	400–1000	100 *, ¥	5.5
Fink et al. [14]	111	360	10	430–950	100 *, ¥	5.5
Song et al. [22]	55	36	10	430–950	71.4 *, α	25
Monheit et al. [16]	1257	1612	10	430–950	98.2 *	9.5
Nagaoka et al. [11]	97	134	124	380–780	96.0 *	87
Stamnes et al. [17]	-	157	10	365–1000	97	97
Stamnes et al. [17]	-	712	10	365–1000	99	93
Hosking et al. [9]	100	52	21	350–950	36 *	100 *
Leon et al. [8]	61	76	116	450–950	87.5/100 *	100
Proposed	61	76	125	450–950	100	80/100 *

## Data Availability

The data presented in this study are available on request from the corresponding author. The data are not publicly available due to patient confidentiality.
